# Characterization of the zinc metalloprotease of *Streptococcus suis* serotype 2

**DOI:** 10.1186/s13567-018-0606-y

**Published:** 2018-10-29

**Authors:** Audrey Dumesnil, Jean-Philippe Auger, David Roy, Désirée Vötsch, Maren Willenborg, Peter Valentin-Weigand, Pyong Woo Park, Daniel Grenier, Nahuel Fittipaldi, Josée Harel, Marcelo Gottschalk

**Affiliations:** 1 Swine and Poultry Infectious Diseases Research Center (CRIPA), Department of Pathology and Microbiology, Faculty of Veterinary Medicine, University of Montreal, Saint-Hyacinthe, QC Canada; 2 Groupe de recherche sur les maladies infectieuses en production animale (GREMIP), Department of Pathology and Microbiology, Faculty of Veterinary Medicine, University of Montreal, Saint-Hyacinthe, QC Canada; 3 Institute for Microbiology, University of Veterinary Medicine Hannover, Hannover, Germany; 4 Boston Children’s Hospital, Harvard Medical School, Boston, MA USA; 5 Oral Ecology Research Group, Faculty of Dentistry, Laval University, Quebec City, QC Canada; 6 Public Health Ontario Laboratory Toronto, and Department of Laboratory Medicine and Pathobiology, University of Toronto, Toronto, ON Canada

## Abstract

*Streptococcus suis* is a swine pathogen and zoonotic agent responsible for meningitis and septic shock. Although several putative virulence factors have been described, the initial steps of the *S. suis* pathogenesis remain poorly understood. While controversial results have been reported for a *S. suis* serotype 2 zinc metalloprotease (Zmp) regarding its IgA protease activity, recent phylogenetic analyses suggested that this protein is homologous to the ZmpC of *Streptococcus pneumonia*e, which is not an IgA protease. Based on the previously described functions of metalloproteases (including IgA protease and ZmpC), different experiments were carried out to study the activities of that of *S. suis* serotype 2. First, results showed that *S. suis*, as well as the recombinant Zmp, were unable to cleave human IgA_1_, confirming lack of IgA protease activity. Similarly, *S. suis* was unable to cleave P-selectin glycoprotein ligand-1 and to activate matrix metalloprotease 9, at least under the conditions tested. However, *S. suis* was able to partially cleave mucin 16 and syndecan-1 ectodomains. Experiments carried out with an isogenic Δ*zmp* mutant showed that the Zmp protein was partially involved in such activities. The absence of a functional Zmp protein did not affect the ability of *S. suis* to adhere to porcine bronchial epithelial cells in vitro, or to colonize the upper respiratory tract of pigs in vivo. Taken together, our results show that *S. suis* serotype 2 Zmp is not a critical virulence factor and highlight the importance of independently confirming results on *S. suis* virulence by different teams.

**Electronic supplementary material:**

The online version of this article (10.1186/s13567-018-0606-y) contains supplementary material, which is available to authorized users.

## Introduction

*Streptococcus suis* is a swine pathogen responsible for cases of meningitis, arthritis, endocarditis, and sudden death in post-weaned piglets. It is responsible for substantial economic losses to the porcine industry and it also represents a serious problem due to the routine use of antimicrobials in the field in attempts to control the infection [1]. It is also an emerging zoonotic agent causing meningitis and septic shock in individuals associated with the swine/pork industry in Western countries or among the general population in some Asian countries [1]. A total of 35 capsular-based serotypes have been reported, with certain of these having recently been described as belonging to other bacterial species [2]. Of the different serotypes, serotype 2 is the most frequently isolated from diseased pigs and humans worldwide [1]. Serotype 2 strains differ greatly in terms of virulence potential and geographic distributions and it can be further classified into different sequence types (ST) based on the multilocus sequence typing (MLST) scheme. Indeed, most virulent strains isolated in Europe and Asia belong to ST1, whereas ST25 and ST28 strains, considered as less virulent, are mainly present in North America [3]. ST7 serotype 2 strains, responsible for at least two major outbreaks of human *S.* *suis* infections in China, are considered highly virulent [4].

The initial mechanisms involved in *S.* *suis* colonization of the host remain poorly known, with the pathogen being able to survive in the tonsils of swine for long periods of time [5]. *S.* *suis* has been described to colonize and interact with epithelial cells and mucus of the host upper respiratory tract in order to reach the bloodstream, where it resists phagocytosis and killing [5]. Replication in blood and systemic dissemination allow *S.* *suis* to subsequently invade the central nervous system and cause meningitis [6]. Over the years, different bacterial components have been suggested to be involved in the *S.* *suis* pathogenesis, including the capsular polysaccharide, the suilysin, the extracellular protein factor (EF), and the muramidase-released protein [7]. However, controversy continues to persist regarding the role of so-called critical *S. suis* virulence factors [7].

Type A immunoglobulins (IgA) are the predominant immunoglobulin class produced by mucosa-associated lymphoid tissues. They may prevent the adhesion of microorganisms to epithelial cells and consequently facilitate their elimination from the host [5]. In the case of *S.* *suis*, the secretion of an active human IgA_1_ protease, a zinc-dependent metalloprotease (Zmp) encoded by the *iga* gene, has been reported in a serotype 2 ST7 strain [8]. The decreased lethality in pigs following intranasal inoculation of an Δ*iga* mutant strain suggested that mucosal IgAs play a crucial role in resistance to *S.* *suis* invasion and host dissemination [8, 9]. However, this conclusion may be questionable based on three main considerations: firstly, porcine specific or cross-reactive IgAs against *S.* *suis* have never been documented [5]; secondly, no IgA protease activity against human IgAs was detected in any of the *S.* *suis* strains evaluated in a subsequent study [10], and thirdly, in silico amino acid sequence analysis, as well as structural homology comparisons, do not support the notion that the Zmp encoded by gene *iga* can have IgA protease activity. In fact, Zmps have been well described in *Streptococcus pneumoniae* and are classified into four distinct groups: ZmpA (IgA protease), ZmpB, ZmpC, and ZmpD [10]. Similar to *S.* *pneumoniae* Zmps, that of *S.* *suis* is a membrane protein attached by a cell-wall LPXTG-anchored motif that possesses G5 tandem repeats and a M26 protease active site. This catalytic site is characterized by a HEMVH motif, which is a key characteristic of the *S.* *pneumoniae* ZmpC (but not ZmpA) [8, 10]. In accordance, phylogenetic studies have classified the *S.* *suis* Zmp as an homologue of the *S.* *pneumoniae* ZmpC based on genomic sequence similarities [10]. To avoid confusion, we will hereafter refer to the factor as Zmp. Importantly, the gene coding for this protein (*zmp*) was found to be present in several of the genomes of 300 *S.* *suis* strains recently analyzed [11].

*Streptococcus pneumoniae* ZmpC has been described to possesses different activities, which include activation of matrix metalloproteinase 9 (MMP-9) and cleavage of P-selectin glycoprotein ligand-1 (PSGL-1), mucin 16 (MUC16), and syndecan-1 (SDC-1) ectodomains [10, 12–15]. Consequently, given the genetic sequence similarities between Zmps of *S.* *suis* and *S. pneumoniae*, it is possible that this protein could have an important impact on the first steps of *S.* *suis* pathogenesis. Moreover, some of these functions could also play important roles in invasion of the central nervous system [16]. However, the actual role of *S.* *suis* Zmp in the pathogenesis of the infection has not been completely evaluated. Finally, it is still not clear if Zmp (or *S.* *suis* in general) is able to present an IgA protease activity.

In the present study, putative functions as well as the role in virulence of the *S.* *suis* Zmp were studied. We report that *S.* *suis* not only does not cleave human IgA_1_, but it is also unable to cleave PSGL-1 ectodomains or to activate MMP-9. However, Zmp is responsible, at least in part, for the *S.* *suis* cleavage of MUC16 and SDC-1 ectodomains, though this activity does not appear to have a critical impact on *S.* *suis* serotype 2 colonization of the upper respiratory tract nor virulence.

## Materials and methods

### Bacterial strains, plasmids, and growth conditions

The strains and plasmids used in this study are listed in Table 1. The virulent *S.* *suis* serotype 2 strain P1/7 was used for the construction of an isogenic *zmp*-deficient mutant. The *S.* *pneumoniae* strain TIGR4 (American Type Culture Collection, Manassas, VA, USA) was used as a positive control for most activation and cleavage assays. Streptococci were grown in Todd-Hewitt broth (THB) until exponential growth phase or agar (THA) for 18 h (Becton–Dickinson, Sparks, MD, USA) 37 °C with 5% CO_2_, with the exception of the SDC-1 ectodomain cleavage experiment (see below), for which bacteria were used in stationary growth phase as previously described for other pathogens [12, 17, 18]. *Escherichia coli* strains were grown in Luria–Bertani (LB) broth or agar (Becton–Dickinson) at 37 °C. When needed, antibiotics were added at the following concentrations: for *S.* *suis*, spectinomycin at 100 µg/mL; for *E.* *coli*, kanamycin at 25 µg/mL and ampicillin, spectinomycin and carbenicillin at 50 µg/mL (Sigma, Oakville, ON, Canada).

**Table 1:** **Bacterial strains and plasmids used in this study**

Strains/plasmids	General characteristics	Source/references
*Escherichia coli*		
TOP 10	F^−^ mrcA Δ(mrr-hsdRMS-mcrBC) φ80 lacZΔM15 ΔlacX74 recA1 araD139 Δ(ara-leu) 7697 galU galK rpsL (Str^R^) endA1 nupG	Invitrogen
BL21	F^−^ompT hsdS_B_ (r_B_^−^, m_B_^−^) gal dcm rne131 (DE3)	Invitrogen
*Streptococcus suis*		
P1/7	Wild-type, virulent European serotype 2 ST1 strain isolated from pig with meningitis	[56]
Δ*zmp*	Isogenic mutant strain derived from P1/7; in frame deletion of *zmp* gene	This work
compΔ*zmp*	Mutant Δ*zmp* complemented with pMX*zmp* complementation vector	This work
SC84	Highly virulent clonal serotype 2 ST7 strain isolated from the 2005 human outbreak in China	[57]
*Streptococcus pneumoniae*		
TIGR4	Virulent serotype 4 strain isolated from human blood in Norway	ATCC
Plasmids		
PCR2.1	Ap^r^, Km^r^, oriR(f1) MCS oriR (ColE1)	Invitrogen
pSET4s	Thermosensitive vector for allelic replacement in *S.* *suis*. Replication functions of pG + host3, MCS oriR pUC19 lacZ Sp^R^	[20]
pMX1	Replication functions of pSSU1, MCS pUC19 lacZ SpR, malX promoter of *S.* *suis*, derivative of pSET2	[20, 58]
pET101	Ap^r^, pBR322 *ori*, T7 promotor	Invitrogen
p4Δ*zmp*	pSET4 s carrying the construct of *zmp* for allelic replacement	This work
pMX*zmp*	pMX1 complementation vector carrying intact *zmp* gene	This work
pET101*zmp*	pET101 carrying *zmp* gene for protein production	This work

### DNA manipulations

*Streptococcus suis* genomic DNA was extracted using InstaGene Matrix solution (Biorad Laboratories, Hercules, CA, USA). Plasmid minipreparations were performed with the QIAprep Spin Miniprep Kit (Quiagen, Valencia, CA, USA). Restriction and DNA-modifying enzymes were purchased from Fisher Scientific (Ottawa, ON, Canada) and used according to the manufacturer’s recommendations. Oligonucleotide primers (listed in Table 2) were obtained from Integrated DNA Technologies (Coralville, IA, USA). Polymerase chain reactions (PCR) were carried out using the iProof high-fidelity DNA polymerase (BioRad Laboratories, Mississauga, ON, Canada) or the Taq DNA polymerase (New England Biolabs, Ipswich, MA, USA). Amplification products were purified using the QIAquick PCR Purification Kit (Qiagen) and sequenced with an ABI 310 automated DNA sequencer and the ABI PRISM dye terminator cycle sequencing kit (Applied Biosystems, Foster City, CA, USA).

**Table 2:** **Oligonucleotide primers used in this study**

Primer name	Sequence (5′-3′)	Construct
zmp-ID1	CCTTGGTTTAGATGCCC	p4Δ*zmp*
zmp-ID2	CTGAGACAAGTCCCACT	p4Δ*zmp*
zmp-ID3	CAAGCCTTGATGAACTCTAC	p4Δ*zmp*
zmp-ID4	GCTATTCCTAGCTTCTACCT	p4Δ*zmp*
zmp-ID5	TGTTTCTTTCATCCTTGACAG	p4Δ*zmp*
zmp-ID6	CTTGTTAATGATTATTTAATAAGTTGTTACTCCCTAAAATAG	p4Δ*zmp*
zmp-ID7	TAGGGAGTAACAACTTATTAAATAATCATTAACAAGTTGGTC	p4Δ*zmp*
zmp-ID8	ATCTGGCTCATCCATGAC	p4Δ*zmp*
pMX1-cpec-F	AAATAGTATAGAAACGGCATGCAAGCTTGG	pMX*zmp*
pMX1-cpec-R	TCGGAGGTCCTTTAGCCCGGGTACCGAGCT	pMX*zmp*
zmp-cpec-F	CCAAGCTTGCATGCCGTTTCTATACTATTT	pMX*zmp*
zmp-cpec-R	AGCTCGGTACCCGGGCTAAAGGACCTCCGA	pMX*zmp*
pET101-zmp-F	CACCATGGCTCGATATAACCATGCAATC	pET101*zmp*
pET101-zmp-R	TGGGTTAAAAATCGATGTTCTG	pET101*zmp*

### Construction of the *S. suiszmp*-deficient (Δ*zmp*) mutant and complemented strain

Precise in-frame deletion of the *zmp* gene was achieved using splicing-by-overlap-extension PCR [19]. Overlapping PCR-products generated by PCR were cloned into the plasmid pCR2.1 (Invitrogen), extracted using EcoRI, and cloned into the thermosensitive *E. coli*–*S. suis* shuttle vector pSET4s, as previously described [20]. The resulting mutation vector p4∆*zmp* was electroporated into competent *S. suis* recipient cells. Allelic replacement and deletion of the gene was confirmed by PCR and sequence analysis.

Circular polymerase extension cloning (CPEC) was used for the construction of the complementation vector of *zmp* gene, as described previously [21]. Briefly, the complementation vector pMX1, which possesses a *malQ* inducible promoter, was linearized by PCR and the complete *zmp* gene amplified. Overhanging primers were used to ligate the gene into the linearized pMX1. The construction was then purified and transformed into electrocompetent *E. coli* MC1061. The complemented Δ*zmp* (compΔ*zmp*) mutant was obtained by transformation into electrocompetent *S.* *suis* Δ*zmp*. Obtention of compΔ*zmp* was confirmed by PCR and DNA sequencing analysis.

Absence of Zmp expression in the Δ*zmp* mutant and complementation of *zmp* gene were confirmed by immunoblot as previously described [22], using antisera from rabbits immunized with the recombinant Zmp protein (see below). Expression of a capsular polysaccharide in the *S. suis* wild-type strain as well as in both the Δ*zmp* mutant and compΔ*zmp* complemented strains (already described as a major virulence factor [23]) was confirmed by hydrophobicity and coagglutination assays using serotype 2 antiserum, as previously described [24]. For the hydrophobicity assay, 10 mL overnight cultures were centrifuged, washed, and resuspended in phosphate-urea-magnesium sulfate buffer (96 mM K_2_HPO_4_, 53 mM KH_2_PO_4_, 30 mM urea, and 0.8 mM MgSO_4_) at an optical density (OD) 660 nm of 0.6. Four hundred microliter of *n*-hexadecane (Sigma) was then add to 3 mL of the bacterial suspensions, which were vortexed for 2 min, and the OD of the aqueous phase measured. The percentage of hydrophobicity was determined using the following equation: (initial OD − final OD)/initial OD × 100%.

### Expression and purification of the recombinant Zmp protein

The region corresponding to the *zmp* gene, excluding the region encoding the LPVTG motif (to facilitate the purification of the recombinant protein from *E. coli*), was amplified by PCR and directly cloned into the pET101 vector (Invitrogen), which possesses a C-terminal His-tag, according to the manufacturer’s instructions. Protein production was induced in the *E. coli* BL21 (DE3) strain using 0.5 mM IPTG for 4 h, after which cells were lysed by sonication. Cell lysates were used to purify the recombinant Zmp protein by His-Bind Resin Chromatography Kit (Novagen, Madison, WI, USA) according to manufacturer’s instructions and dialysed. Protein purity was confirmed by SDS-PAGE and Western blot using an anti-His-tag antibody (R&D Systems, Minneapolis, MN, USA). The purified recombinant protein was concentrated using Amicon Ultra-15 (Millipore, Billerica, MA, USA) and protein quantification was evaluated using the Pierce Bicinchoninic Acid Protein Assay Kit (Thermo Scientific). A mono-specific polyclonal hyperimmune serum was produced in rabbits using the purified Zmp protein as previously described [25].

### IgA protease cleavage assay

The IgA protease cleavage assay was performed as previously described with some modifications [10, 14]. Briefly, 5 µg of human myeloma IgA_1_ (Calbiochem/EMD Millipore, San Diego, CA, USA) were incubated with either purified recombinant Zmp (100 µg), 1 × 10^8^ CFU/mL of live washed *S. pneumoniae* (positive control), 1 × 10^9^ CFU/mL of live washed *S. suis* P1/7 strain or supernatants (non-concentrated or concentrated up to 10X) of each bacterial species for 16 h at 37 °C. Using similar conditions, the clonal ST7 strain SC84, responsible for the 2005 Chinese human outbreak [26], was also tested. Bacterial supernatants were filtrated and concentrated up to 10X using the Thermo Savant DNA120 Speedvac (Thermo Fisher). Samples were then separated on 7.5% SDS-PAGE under denaturing conditions and transferred onto nitrocellulose membrane (Bio-Rad). The size of uncleaved and cleaved human IgA_1_ was detected using Western blot with mouse monoclonal B3506B4 anti-human IgA_1_ Fc horseradish peroxidase (HRP)-conjugated antibodies diluted 1:500 (Abcam, Cambridge, MA, USA) and detected using HyGLO Chemiluminescent HRP Antibody Detection Reagent (Denville Scientific, Metuchen, NJ, USA).

### Activation of matrix metalloprotease-9

MMP-9 activation assay was performed as previously described with some modifications [10, 14]. Briefly, 10 ng of recombinant human pro-enzyme MMP-9 (Calbiochem/EMD Millipore) were incubated with recombinant Zmp (100 µg), *S. suis* or *S. pneumoniae* (washed bacteria and supernatants as described above) for 1 h at 37 °C. Samples were then separated on 10% zymogram gels containing gelatin B (Sigma) under non-denaturing conditions. Results were visualized after Coomassie Blue R250 staining (Bio-Rad).

### Cleavage of P-selectin glycoprotein ligand-1 ectodomains

The PSGL-1 cleavage assay was performed as previously described with some modifications [15]. Briefly, 10 ng of recombinant human PSGL-1/CD162 Fc (R&D Systems, Minneapolis, MN, USA) were incubated with recombinant Zmp (100 µg), *S. suis* or *S. pneumoniae* (washed bacteria and supernatants, as described above) for 1 h at 37 °C. Samples were then separated on 7.5% SDS-PAGE under denaturing conditions and transferred to polyvinyl difluoride (PVDF) membrane (EMD Millipore). The size of cleaved and uncleaved human PSGL-1/Fc was detected using Western blot with mouse monoclonal anti-human CD162 antibodies, clones KPL-1 and PL2, diluted 1:1000 (Becton–Dickinson Biosciences; MBL International Corporation, Woburn, MA, USA) and horseradish peroxidase conjugated (HRP) goat anti-mouse antibodies (Jackson ImmunoResearch, West Grove, PA, USA). Detection was achieved by using HyGLO Chemiluminescent HRP Antibody Detection Reagent (Denville Scientific).

### Cleavage of mucin 16 ectodomains

The MUC16 ectodomain cleavage assay was performed as previously described with some modifications [13, 27]. The HeLa (CCL-2) cell line was obtained from the ATCC and incubated at 37 °C with 5% CO_2_. Once confluent, cells were trypsinized, transferred to 24-well tissue culture plates (Costar) at a concentration of 7.5 × 10^4^ cells/mL, and further incubated to confluence from 2 to 3 days (approximately 10^5^ cells/well) [28]. Cells were then washed twice with phosphate-buffered saline (PBS), pH 7.3, before being treated with recombinant Zmp (100 µg), *S. suis* or *S. pneumoniae*, which was used as a positive control (washed bacteria and supernatants as described above). In a parallel study, *S. suis*Δ*zmp*-concentrated (3X) supernatants supplemented or not with 100 µg of recombinant Zmp and *S.* *suis* compΔ*zmp* concentrated (3X) supernatants were also tested. Absence of toxicity was verified using the CytoTox 96^®^ Assay Kit (Promega, Madison, WI, USA). Cells were incubated for 4 h at 37 °C, after which supernatants were recovered and concentrated two-fold with Thermo Savant DNA120 Speedvac (Thermo Fisher). Cleaved MUC16 ectodomains were determined using DOT-blot on nitrocellulose membrane with 1:100 mouse anti-human CA125 antibody (M11) (Thermo Fisher) and a 1:4000 HRP conjugated goat anti-mouse antibody (Jackson ImmunoResearch). Detection was achieved by using the HyGLO Chemiluminescent HRP Antibody Detection Reagent (Denville Scientific) and signals analysed using ImageJ.50i software (Wayne Rasband, National Institutes of Health, Bethesda, MD, USA).

### Cleavage of syndecan-1 ectodomains

SDC-1 ectodomains cleavage assay was performed as previously described with some modifications [12, 17, 18]. Briefly, NMuMG cell line, derived from a mouse mammary gland, was propagated as previously described [12, 17, 18]. One-day post-confluent NMuMG cells in 96-well tissue culture plates were washed with culture medium and then incubated with 10% concentrated filtered supernatants (10X) from an overnight culture of *S. suis*, *S. suis* Δ*zmp*, *S. suis* compΔ*zmp* for 4 h at 37 °C. Absence of toxicity was verified as described above. Cells were also incubated with recombinant Zmp (100 µg) or 10% *S. suis*Δ*zmp*-concentrated supernatant (10X) supplemented with 100 µg of recombinant Zmp protein. Phorbol 12-myristate 13-acetate (PMA; Sigma) was used as a positive control at a concentration of 1 µM. After incubation of 4 h, conditioned media were collected and acidified as previously described [12, 17, 18]. Samples were dot-blotted on Immobilon Ny+ (Millipore) and shed ectodomains were quantified by immunoblotting using 281-2 rat anti-mouse CD138 antibodies. The intensity of dots was quantified by ImageJ.50i software.

### Adhesion to primary porcine bronchial epithelial cells

Primary porcine bronchial epithelial cells (PBEC) were isolated from lungs obtained from 5 to 6 month old healthy pigs as previously described [29] and differentiated in air–liquid-interface medium (ALI medium), which consists of 50% Dulbecco’s Modified Eagle Medium (DMEM; Life Technologies) and 50% Bronchial Epithelial Growth Medium [BEGM; constituted of bronchial epithelial cell basal medium (BEBM; Lonza) supplemented with several required additives (Sigma)], as previously described [30]. After 4 weeks of differentiation, cells were infected from the apical side, as previously described [29], with approximately 10^7^ CFU/filter of *S.* *suis* strain P1/7 (WT) or Δ*zmp* mutant. Absence of toxicity was verified as described above. Adhesion was analyzed using immunofluorescence microscopy and quantified as previously described [29]. Briefly, this was done semi-quantitatively using a TCS SP5 confocal laser-scanning microscope (Leica) equipped with HCX PL APO CS 20 × 0.7 DRY UV. Image stacks with a z-distance of 0.5 µm per plane were merged and brightness/contrast was adjusted using the ImageJ/Fiji. Area of epithelial cells positive for adherent green fluorescent bacteria was quantified in four randomly selected areas for each treatment using analySIS^®^ 3.2 software (Soft Imaging System GmbH, Münster, Germany).

### *S. suis*Δ*zmp* virulence evaluation in mouse models of infection

In a first experiment, 20 six-week-old CD-1 mice (Charles River, Kingston, ON, Canada) were infected via the intranasal route as previously described [31]. One hour prior to infection, mice were anaesthetized via inhalation of isoflurane (Pharmaceutical Partners of Canada, Richmond Hill, ON, Canada) and pre-treated with 12.5 µL of 1% acetic acid placed in each nostril. Mice were then infected with 8 × 10^9^ CFU of the wild-type strain or Δ*zmp* strain (*n* = 10). The inoculum was applied in two drops of 20 µL placed in each nostril. Alongside, another 20 six-week-old CD-1 mice (Charles River) were with 3 × 10^7^ CFU of the wild-type strain or Δ*zmp* strain (*n* = 10) via the intraperitoneal route, as previously described [32].

For both experiments, health and behavior were monitored at least thrice daily until 72 h post-infection and twice thereafter until the end of the experiment (14 days post-infection) for the development of clinical signs of sepsis, such as depression, swollen eyes, rough hair coat, and lethargy. Mice were also monitored for the development of clinical signs of meningitis. Mice were immediately euthanized upon reaching endpoint criteria using CO_2_ followed by cervical dislocation. No mice died before meeting endpoint criteria and all surviving mice were euthanized as described above at the end of the experiment.

### Evaluation of the colonization capacity and virulence of the *S.* *suis*Δ*zmp* in a porcine model of infection

Four-week old piglets (*n* = 12) from a herd with no recent cases of *S. suis* disease were used. Animals were randomly separated into two rooms upon arrival (6 animals per room) and the trachea and tonsils were swabbed to confirm absence of serotype 2 European-like strains using a multiplex PCR targeting the *epf* gene that codes for the EF [33], usually absent in North American serotype 2 strains [3], and *cps*2*j* gene, coding for the capsular polysaccharide of serotype 2 strains. The *S.* *suis* wild-type serotype 2 strain P1/7 (which is *epf* and *cps*2*j* positive) and Δ*zmp*-deficient mutant were cultured as previously described [32] to obtain a final concentration of 2 × 10^10^ CFU/mL. Intranasal infections were carried out as previously described with some modifications [34]. Pigs were inoculated with 2 mL of 1% acetic acid per nostril 2 h prior to infection with 2 mL per nostril of either the wild-type strain or Δ*zmp* mutant. Trachea and tonsils were swabbed using a catheter (Medline, Waukegan, IL, USA) or a brush (Medical Packaging Corporation, Camarillo, CA, USA), respectively, immediately prior to infection, 24 h after infection, and every 2 days thereafter. Samples were placed in PBS supplemented with 0.1% bovine serum albumin and immediately cultured. Serial dilutions (10^0^–10^−6^) were plated on Colombia Agar (Oxoid, Hampshire, UK) supplemented with 5% defibrinated sheep blood (Cedarlane, Burlington, ON, Canada), and *Streptococcus* Selective Reagent SR0126 (Oxoid). After incubation for 18 h at 37 °C with 5% CO_2_, plates containing between 30 and 300 colonies were selected. Suspected alpha-hemolytic colonies were enumerated and 10 *S.* *suis*-like colonies/plate were sub-cultured in order to perform the *epf* and *cps2j* multiplex PCR [33].

Clinical signs of pigs were monitored throughout the experiment. Nine days post-infection, remaining animals were euthanized. Liver, spleen, and brain samples were collected upon euthanasia. All samples were evaluated for carriage of *S.* *suis* serotype 2 *epf*^+^ as described above. *S.* *suis* serotype 2 strains recovered from euthanized animals infected with the mutant strain were monitored for absence of the *zmp* gene by PCR.

### Statistical analyses

Data are expressed as mean ± standard error of the mean (SEM). Significant differences were determined using the t-test, Mann–Whitney Rank sum test or one-way ANOVA, where appropriate. Each test was repeated in at least three independent experiments. For in vivo virulence experiments, survival was analyzed using the LogRank test. *p* < 0.05 was considered statistically significant.

## Results

### Confirmation of the Zmp-deficient and complemented *S. suis* mutants

**Figure 1:**
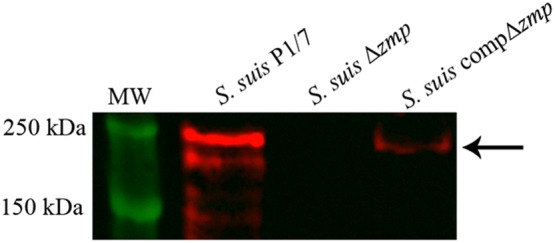
**Expression of** ***S. suis*** **Zmp protein is abrogated in the** ***zmp*-deficient mutant.** Culture supernatants of the *S. suis* wild-type strain P1/7 (lane 2), *zmp*-deficient mutant (Δ*zmp*) (lane 3), and complemented strain (compΔ*zmp*) (lane 4) were separated by SDS-PAGE and transferred onto nitrocellulose membrane. The Zmp protein was detected using a rabbit antiserum against Zmp. The black arrow corresponds to the Zmp protein and MW to molecular weight ladder (lane 1).

A Western blot using a rabbit anti-Zmp serum confirmed production of Zmp protein in the *S.* *suis* wild-type strain P1/7, which was abrogated following in-frame deletion of the *zmp* gene in the mutant strain (Figure 1). Complementation of *zmp* gene (*S.* *suis* compΔ*zmp*) restored production of Zmp, albeit expression was lower than that of the wild-type parent strain (Figure 1). Moreover, gene deletion and complementation had no impact on growth of the mutant in comparison with the wild-type strain (results not shown). The mutant and complemented strains were shown to be well-encapsulated by the hydrophobicity test and the clear positive reaction by coagglutination using serotype 2 anti-serum (results not shown).

### *S. suis* is unable to cleave human IgA_1_

**Figure 2:**
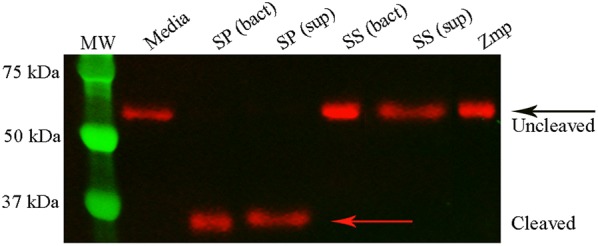
***S.*** ***suis*** **is unable to cleave human IgA**_**1**_. Purified recombinant Zmp protein (100 µg), washed bacteria (bact) or bacterial supernatant (sup) from *S.* *suis* (SS) or *S.* *pneumoniae* (SP) were incubated with human IgA_1_ for 16 h and reactions separated by SDS-PAGE. Cleaved IgA_1_ (red arrow) by washed *S.* *pneumoniae* bacteria or bacterial supernatant (lanes 3 and 4) and uncleaved IgA_1_ (black arrow) by washed *S.* *suis* bacteria (lane 5), bacterial supernatant (10X, lane 6), and purified Zmp protein (lane 7) were visualized using a specific antibody against human IgA_1_. MW corresponds to the molecular weight ladder (lane 1) and media correspond to untreated human IgA_1_ (lane 2).

In order to confirm the ability of *S.* *suis* and Zmp to cleave IgAs, recombinant Zmp, bacterial suspensions (bact) or bacterial supernatants (sup; either non-concentrated or concentrated) were incubated with human IgA_1_. For *S.* *pneumoniae* strain TIGR4 (SP), used as a positive control, an IgA protease activity was clearly observed (Figure 2). On the contrary, recombinant *S.* *suis* Zmp was unable to cleave human IgAs (Figure 2). In addition, the virulent strain P1/7 of *S.* *suis* used in this study (both whole live washed bacteria and culture supernatants) was completely unable to cleave human IgAs (Figure 2). Similarly, *S.* *suis* ST7 strain SC84 did not possess any IgA protease activity (Additional file 1).

### *S. suis* does not activate MMP-9

**Figure 3:**
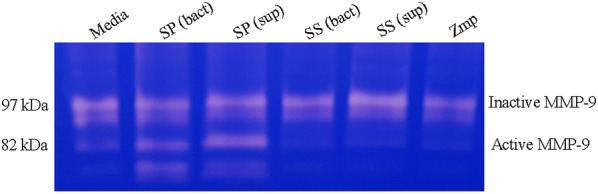
***S.*** ***suis*** **does not activate matrix metalloprotease-9 (MMP-9).** Pro-enzyme MMP-9 was incubated for 1 h with purified recombinant Zmp protein (100 µg), washed bacteria (bact) or bacterial supernatant (sup) from *S.* *suis* (SS) or *S. pneumoniae* (SP). Samples were separated in non-denaturing conditions on a gelatin B zymogram. Coomassie coloration shows a second gelatinolytic band for washed *S.* *pneumoniae* bacteria or bacterial supernatants (lane 2 and 3) corresponding to activated MMP-9, while no second band was observed for media (lane 1) washed *S.* *suis* bacteria (lane 4), bacterial supernatant (10X, lane 5) or purified Zmp protein (lane 6).

To assess the capacity of *S.* *suis* and Zmp to activate MMP-9, the enzyme pro-form (97 kDa) was incubated with recombinant Zmp, bacteria (bact) or bacterial supernatant (sup; either non-concentrated or concentrated). MMP-9 activation was then visualized using gelatin B zymogram, wherein absence of color after Coomassie staining indicates presence of a gelatinase activity. As observed in Figure 3, *S.* *pneumoniae* strain TIGR4 (SP), used as a positive control, was able to activate MMP-9 since an additional clear band corresponding to the gelatinolytic activity of activated MMP-9 appeared on the gel at a molecular weight smaller than 82 kDa. However, no activation of MMP-9 was observed in the presence of recombinant Zmp or with *S.* *suis* (SS) with either whole live washed bacteria or bacterial supernatants (Figure 3).

### *S. suis* is unable to cleave PSGL-1 ectodomains

**Figure 4:**
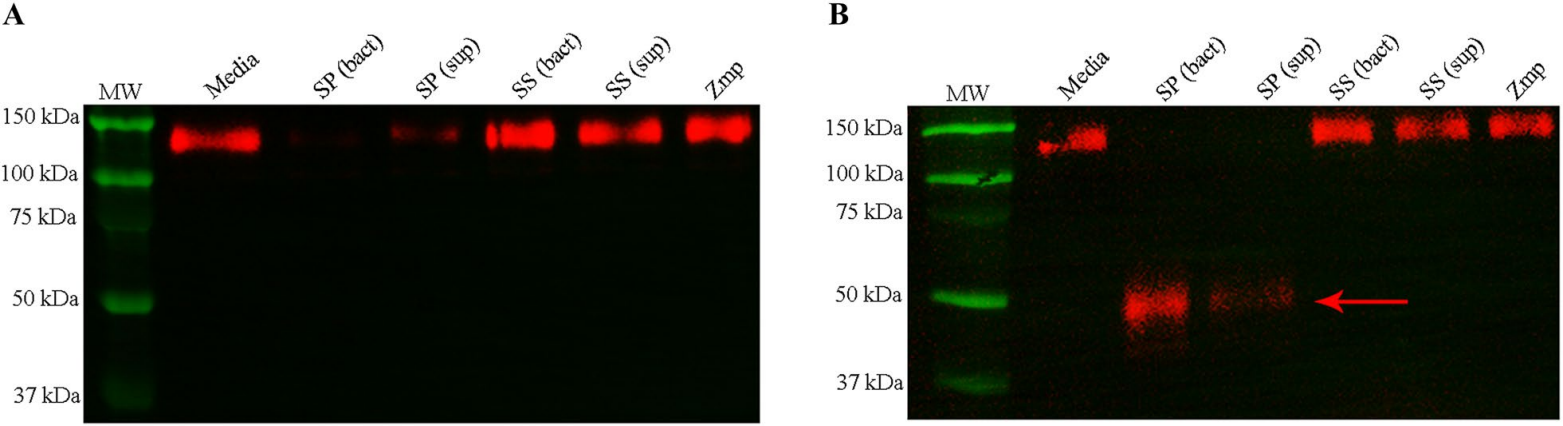
***S.*** ***suis*** **is unable to cleave P-selectin glycoprotein ligand-1 (PSGL-1) ectodomains.** Purified recombinant Zmp protein (100 µg), washed bacteria (bact) or bacterial supernatant (sup) from *S.* *suis* (SS) or *S.* *pneumoniae* (SP) were incubated with PSGL-1/fc for 1 h. Samples were separated by SDS-PAGE and transferred onto PVDF membranes, which were incubated with KPL-1 (**A**) or PL2 (**B**) antibody. No recognition of PSGL-1 by the KPL-1 antibody and visualization of lower molecular weight band with PL2 antibody were observed for washed *S.* *pneumoniae* bacteria and bacterial supernatant (lanes 3 and 4), which is expected following PSGL-1 ectodomain cleavage. There was no change in the recognition by KPL-1 or in the molecular weight visualized with PL2 for PSGL-1 treated with washed *S.* *suis* bacteria (lane 5), bacterial supernatant (10X, lane 6) or the purified recombinant Zmp protein (lane 7). MW corresponds to the molecular weight ladder (lane 1) and media correspond to untreated PSGL-1 (lane 2).

In order to evaluate the ability of *S.* *suis* and Zmp to cleave PSGL-1 ectodomains, recombinant Zmp, whole bacteria (bact) or bacterial supernatants (sup) were incubated with PSGL-1/fc. To visualize cleavage, two different antibodies were used, whether KPL-1 and PL-2, which recognize the N-terminal epitope of PSGL-1 and a membrane-proximal epitope, respectively [35]. As expected [15], the *S.* *pneumoniae* strain TIGR4 (SP), used as a positive control, cleaved PSGL-1 ectodomains (Figure 4). It was observed that the reactivity of KPL-1 antibody with PSGL-1/fc diminished as a result of the N-terminal epitope of PSGL-1/fc being cleaved (Figure 4A). Meanwhile, PL2 still recognized the degradation products of PSGL-1/Fc (Figure 4B). This was expected since the *S.* *pneumoniae* ZmpC removes the N-terminus of PSGL-1 required for recognition by KPL1 with PSGL-1/Fc, since KPL1 recognizes a N-terminal epitope of PSGL-1 [35]. On the other hand, PL2 can still recognize multiple degradation products between 150 and 25 kDa since it recognizes a membrane-proximal epitope [35]. Meanwhile, recombinant Zmp, *S.* *suis* (SS) bacterial cells as well as bacterial supernatants were unable to cleave PSGL-1 ectodomains (Figure 4).

### Zmp is partially implicated in the capacity of *S.* *suis* to cleave MUC16 ectodomains

**Figure 5:**
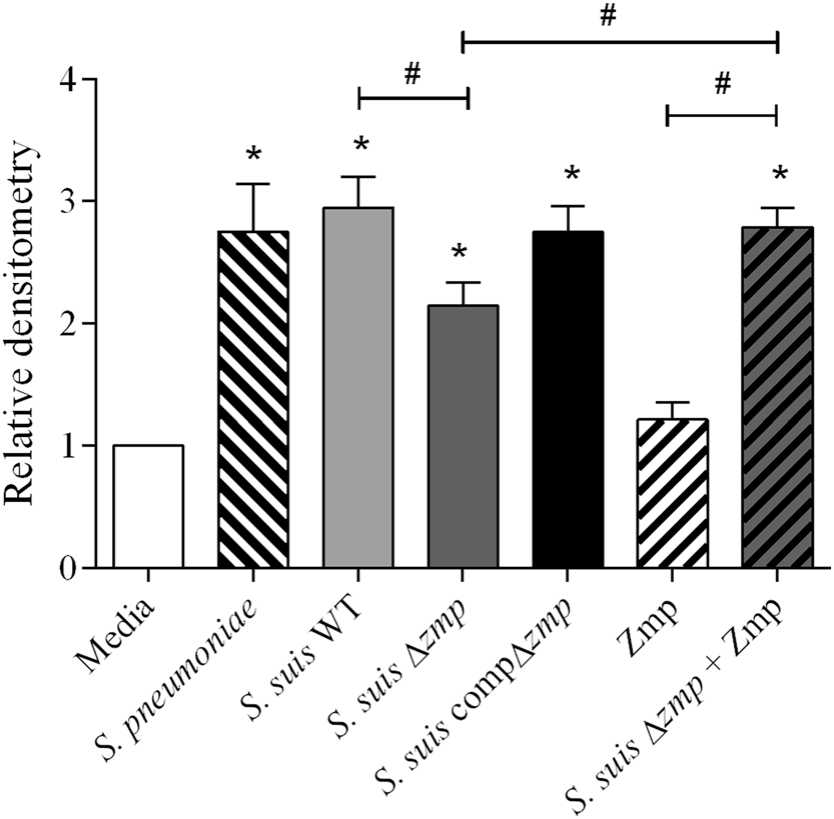
**Zmp is partially implicated in mucin 16 (MUC16) ectodomain cleavage by** ***S. suis***. Relative densitometry of HeLa cell culture medium conditioned with non-concentrated *S.* *pneumoniae* supernatant, Zmp purified recombinant protein, *S.* *suis* P1/7 (WT) or *S.* *suis* Δ*zmp* supernatant (3X) supplemented or not with 100 µg of recombinant Zmp protein. Samples were blotted on a nitrocellulose membrane and incubated with anti-human CA125 antibodies. Compared to the untreated cells, *S.* *suis* WT strain P1/7 supernatant cleaved MUC16 ectodomains. This activity was significantly reduced when using the supernatant of the Δ*zmp* strain (*p* = 0.017). Supernatant of the compΔ*zmp* significantly increased cleavage when compared to the Δ*zmp* strain. Addition of 100 µg of Zmp protein to the supernatant of the Δ*zmp* strain also restored WT phenotype activity. Data represent the mean ± SEM from at least three independent experiments. *(*p* < 0.05) indicates a significant difference between treated groups and the medium; ^#^(*p* < 0.05) between treatment groups.

A treatment of HeLa cells with live washed bacteria or bacterial supernatants concentrated 10X was found to be cytotoxic (results not shown). Thus, *S.* *pneumoniae* non-concentrated supernatant (positive control) and *S.* *suis* supernatant (either non-concentrated or concentrated 3X) were used to perform the MUC16 ectodomain cleavage assay. As shown in Figure 5, *S.* *pneumoniae* non-concentrated supernatant cleaved MUC16 ectodomains. However, only after being concentrated 3X was *S.* *suis* supernatant from wild-type strain P1/7 able to cleave MUC16 ectodomains. As such, Zmp activity of *S.* *suis* seems to be less strong than that of *S. pneumoniae*. Furthermore, deletion of the *zmp* gene significantly decreased the ability of *S.* *suis* supernatant to cleave MUC16 ectodomains (*p* = 0.017), but not to the level of the negative control. However, *S.* *suis* recombinant Zmp alone is unable to cleave MUC16 ectodomains (*p* = 0.304). Meanwhile, complementation of *zmp* gene in *S.* *suis* (compΔ*zmp*) partially restored the wild-type phenotype, in comparison with the isogenic mutant (*p* = 0.067), but was still less active than the wild-type strain. However, complementation of the *S.* *suis*Δ*zmp* supernatant by addition of 100 µg of recombinant Zmp completely restored the wild-type phenotype (*p* = 0.907). Residual MUC16 ectodomain shedding ability when using the *S.* *suis*Δ*zmp* supernatant suggests that other factors are potentially responsible for cleavage by *S.* *suis*.

### Zmp is partially implicated in the capacity of *S.* *suis* to cleave SDC-1 ectodomains

**Figure 6:**
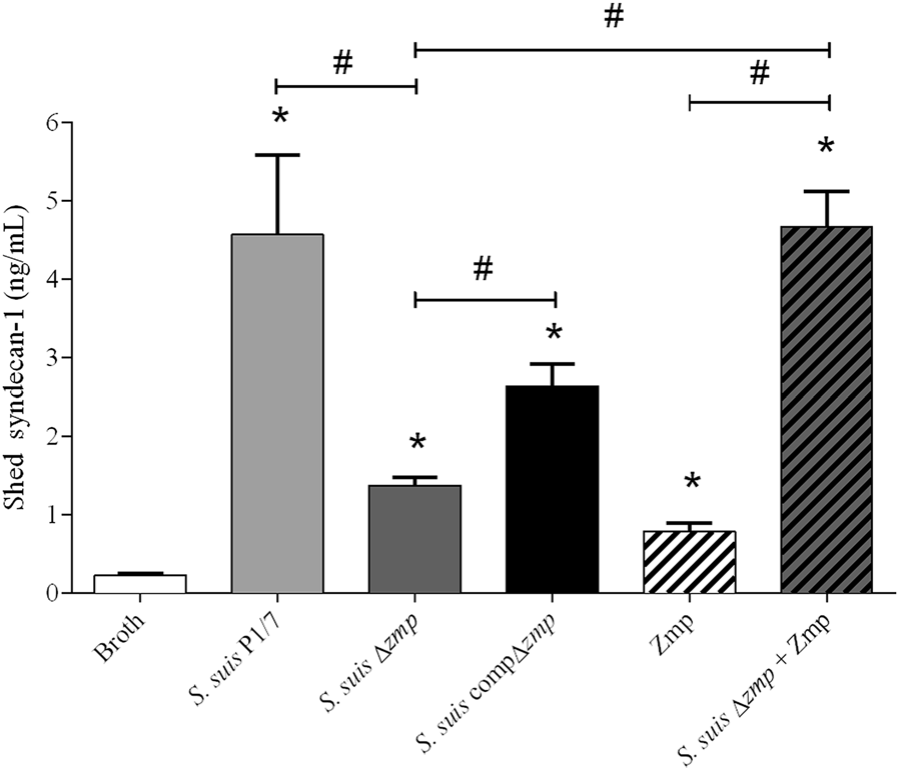
**Zmp is implicated in SDC-1 ectodomain cleavage.** The concentration of SDC-1-cleaved ectodomains in NMuMG cell culture medium was measured after treatment with 100 µg of Zmp protein, 10X concentrated supernatants of either *S.* *suis* wild-type P1/7 (WT), *S.* *suis* Δ*zmp* (supplemented or not with 100 µg of recombinant Zmp) or *S.* *suis* compΔ*zmp*. Samples were blotted on Immobilon Ny+ and incubated with anti-mouse SDC-1 (281-2) antibodies. The capacity of the Δ*zmp* mutant to cleave SDC-1 ectodomains was significantly reduced in comparison to the WT strain, while the complementation of *zmp* gene restored WT phenotype. Recombinant Zmp protein also cleaved SDC-1 ectodomains. This activity was amplified when Zmp protein was incubated with *zmp*-deficient mutant culture supernatant. The concentration of shed SDC-1 ectodomains was determined using SDC-1 ectodomains purified from NMuMG cells as standards, as previously described [12]. Data represent the mean ± SEM from at least three independent experiments. *(*p* < 0.05) indicates a significant difference between treated groups and the medium; ^#^(*p* < 0.05) between treated groups.

NMuMG cells treated with 100 µg of recombinant Zmp or *S.* *suis* bacterial supernatants were used to assess SDC-1 ectodomain cleavage. Supernatant concentrated 10X (but not non-concentrated or concentrated 3X; data not shown) from *S.* *suis* wild-type strain P1/7 was able to cleave SDC-1 ectodomains (Figure 6). Furthermore, deletion of the *zmp* gene significantly decreased this ability using the same supernatant concentration (*p* = 0.024); the SDC-1 cleavage activity was restored by complementation of *zmp* gene in strain *S.* *suis* (compΔ*zmp*) (*p* = 0.030), when compared to the activity of the Δ*zmp* mutant strain (Figure 6). Moreover, even if the level of SDC-1 ectodomains released was lower than that by the wild-type strain, there was no statistical difference between these two groups (*p* = 0.139). In contrast to MUC16 ectodomain shedding experiments, *S.* *suis* recombinant Zmp was also able to cleave SDC-1 ectodomains when used alone (*p* = 0.007) (Figure 6). Moreover, addition of 100 µg of purified recombinant Zmp protein to *S.* *suis*Δ*zmp* mutant supernatant completely restored wild-type phenotype (*p* = 0.024), suggesting, once again, the presence of co-factor(s) in *S.* *suis* supernatant. There was also a residual SDC-1 shedding ability in the *zmp*-deficient mutant, suggesting that other factors could potentially be also involved in this activity.

### Zmp is not implicated in adhesion to differentiated primary PBEC

**Figure 7:**
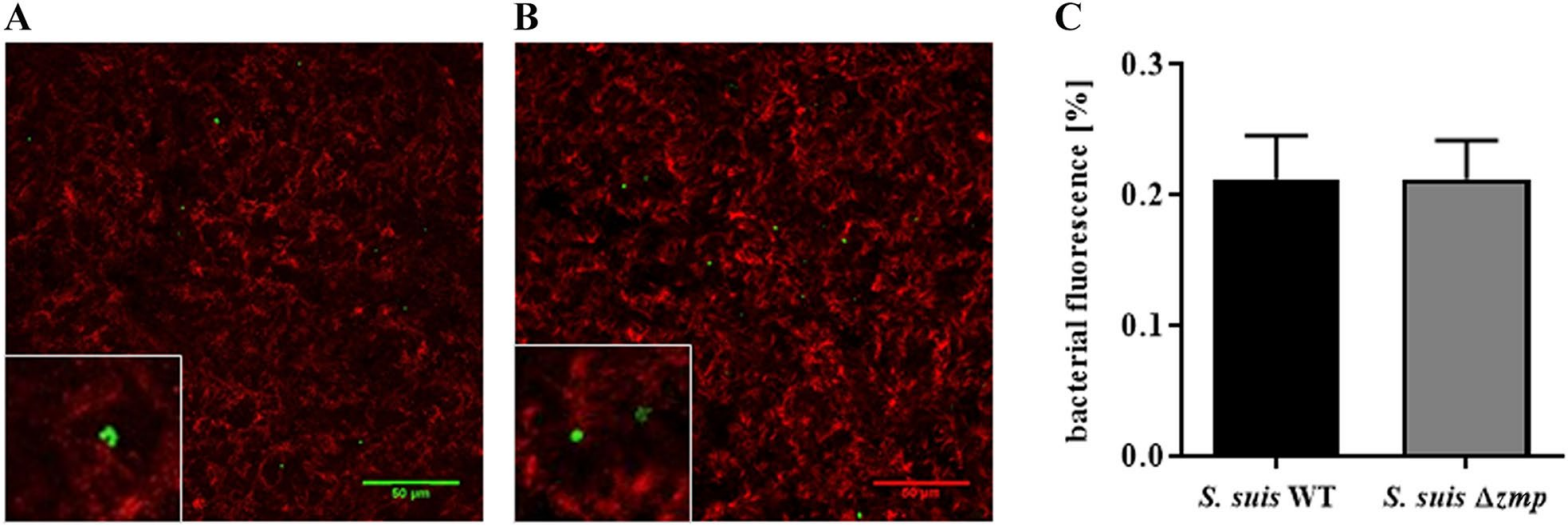
**Zmp is not implicated in adhesion to PBEC under ALI conditions.** Immunofluorescence analyses of PBEC differentiated under ALI conditions and infected for 4 h with **A** *S.* *suis* wild-type P1/7 (WT) and **B** *S.* *suis* ∆*zmp* strain. Ciliated cells were stained for ß-tubulin (red) and streptococci (green). Bars represents 50 µm. **C** Adherent bacteria were quantified by analyzing the epithelial cell surface positive for green fluorescence signal in four randomly chosen areas for each treatment. Results are expressed as mean ± SEM.

The ability of the *S.* *suis* wild-type strain P1/7 and *S.* *suis* Δ*zmp* mutant to colonize primary porcine bronchial epithelial cells was analyzed in ALI cultures with primary PBEC. Cells were infected with 10^7^ CFU/filter (approximate MOI = 25) from the apical side for 4 h and non-adherent bacteria were washed away. Immunofluorescence microscopy showed that the wild-type and Δ*zmp* strains similarly adhered to ciliated epithelial cells (Figures 7A and B). Quantification of adherent bacteria revealed no significant differences in the ability of both strains to colonize PBEC (Figure 7C).

### Zmp is not implicated in colonization of the upper respiratory tract of pigs nor in virulence

**Figure 8:**
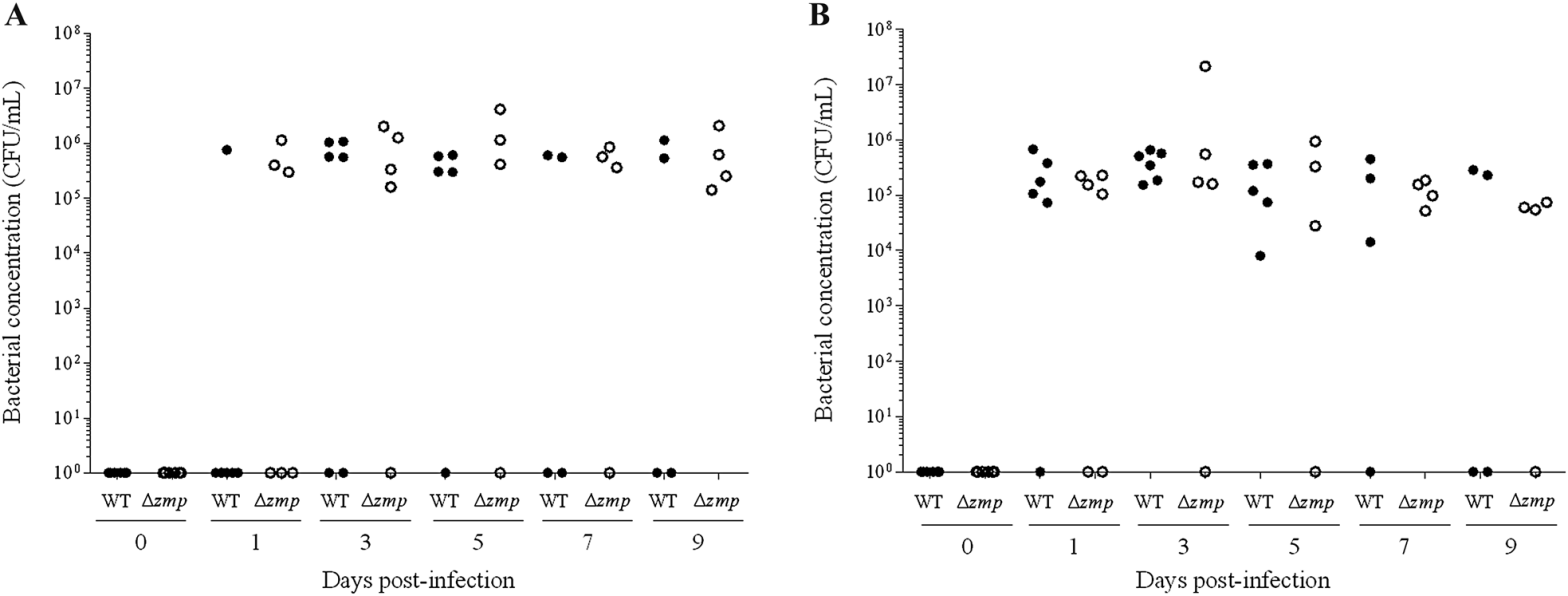
**Presence of Zmp does not influence the colonization potential of** ***S.*** ***suis*** **serotype 2 in a pig model of infection.** Bacterial concentrations recovered in the tonsils (**A**) or trachea (**B**) of piglets infected with either the wild-type strain P1/7 (WT) or its Δ*zmp* mutant. No differences in colonization of the upper respiratory tract of piglets were observed following infection with the Δ*zmp* mutant. Each point represents an individual animal.

**Figure 9:**
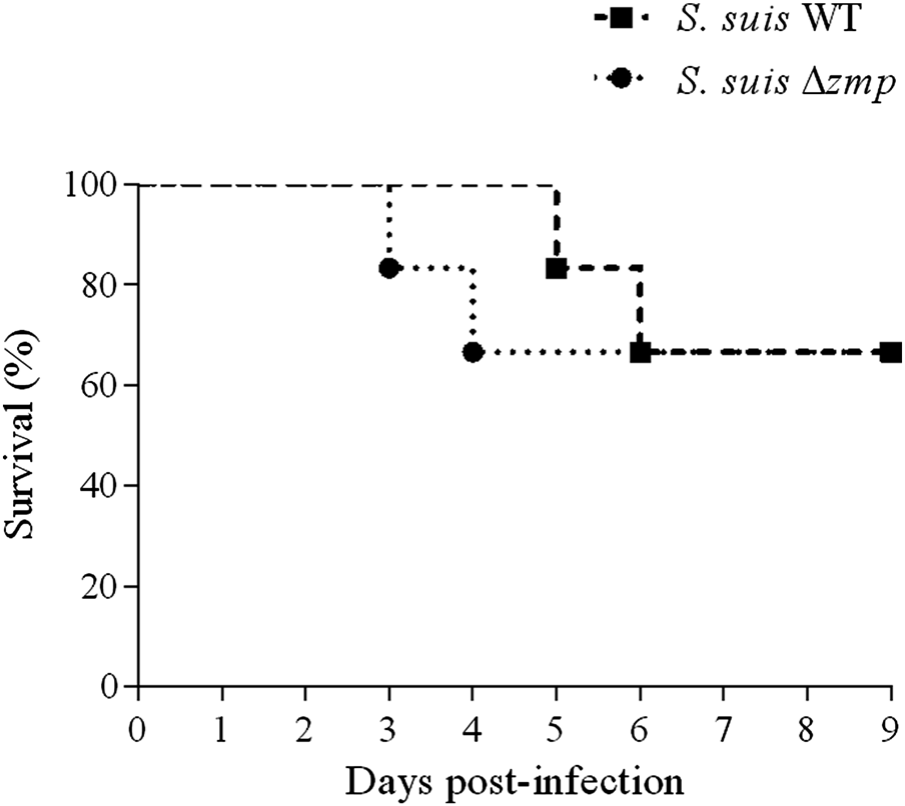
**Zmp does not play a role in virulence of** ***S.*** ***suis*** **serotype 2 using an intranasal pig model of infection.** Survival of 4-week old piglets infected with the wild-type strain P1/7 (WT) or its Δ*zmp* mutant. No significant difference was observed between groups (*p* = 0.839).

Considering the presence of MUC16 in the upper respiratory tract of pigs, and given the previously described function of SDC-1 in adhesion to and invasion of epithelial cells [36, 37], the role of Zmp in the pathogenesis of *S.* *suis* serotype 2 infection, more particularly in colonization, as well as its role as a virulence factor, were evaluated using an intranasal piglet model of *S.* *suis* infection. Animals were divided into two groups and infected with either wild-type strain P1/7 or its *zmp*-deficient mutant. After infection, PCR tests showed presence of *S.* *suis* serotype 2 ST1 (*cps*+, *epf*+) in infected animals. As illustrated in Figure 8A, tonsil colonization was already detectable 24 h post-infection in both groups. As shown in Figure 8B, *S.* *suis* serotype 2 ST1 was also present in the trachea of almost all infected animals in both groups and remained present until day 9. Throughout the experiment, no significant differences were observed between wild-type and Δ*zmp*-infected piglets in both the tonsils and trachea (Figures 8A and B). In addition, results showed no differences in virulence in pigs, since several animals presented severe clinical signs such as arthritis, depression, and meningitis in both groups (*p* = 0.840) (Table 3). Moreover, 33% of piglets in both groups succumbed to infection (Figure 9). Absence of *zmp* gene was confirmed in all *S.* *suis* serotype 2 bacteria recovered from the liver, spleen, and brain of ill animals infected with the mutant strain data not shown). Similar results were also obtained in mice following intranasal (*p *= 0.976) and intraperitoneal (*p *= 0.346) infections (Additional file 2). Following intranasal infection, only 20% of mice presented severe clinical signs, including prostration, depression, and weakness (Additional file 2A). Meanwhile, all intraperitoneally inoculated mice developed severe clinical signs of infection within 5 days post-infection (Additional file 2B).

**Table 3:** **Clinical signs observed in wild-type (WT)- and Δ** ***zmp*** **-infected pigs**

Clinical signs	WT	Δ*zmp*
Fever	4/6	4/6
Respiratory problems	0/6	1/6
Arthritis	3/6	5/6
Neurological problems	2/6	2/6

## Discussion

Increased research over the past decade has led to the identification of a myriad of novel factors putatively involved in the first steps of the *S.* *suis* pathogenesis of infection. In many cases, newly identified factors have been described as “critical” for virulence based on little supporting experimental data, and in some cases, controversial results have been reported in subsequent investigations [7]. One example is the Zmp protease encoded by gene *iga* [8, 10, 38]. Zinc-dependent metalloproteases have been studied relatively extensively in *S. pneumoniae*. Four distinct groups have been described: ZmpA, ZmpB, and ZmpC and ZmpD [10]. *S.* *pneumoniae* ZmpA has been shown to cleave human IgAs, which play an important role in the immunological defense of the respiratory tract and other mucosal surfaces [5], with IgA_1_ representing 90% of the IgA within the human respiratory tract [39]. Indeed, IgA_1_ proteases have been regarded as virulence factors in a variety of pathogens [39–41]. In *S.* *suis*, an IgA protease, with active cleavage effect on human IgA_1_, has been previously described in a virulent *S.* *suis* isolate [8]. In addition, an isogenic mutant defective for the gene encoding this IgA protease was shown to be significantly less virulent than the wild-type strain. Although IgA protease activity of the mutant or parent strain was not experimentally tested, the animal data were interpreted to mean that IgA cleavage in vivo was crucial for virulence [38]. Later, it was shown that immunization with this IgA protease resulted in 100% protection of mice against challenge with a virulent strain [42].

Surprisingly, in a comparative study using different streptococci, Bek-Thomsen et al. reported absence of IgA protease activity in the seven *S.* *suis* strains tested [10]. Consequently, given the controversy surrounding the activity of this metalloprotease in *S.* *suis,* the capacity of *S.* *suis* serotype 2 strain P1/7 (ST1) to degrade human IgA_1_ was evaluated. Results obtained in the present study contradict those originally reported [8] and confirm those published later by Bek-Thomsen et al. [10], with a total absence of IgA protease activity from both *S.* *suis* (non-concentrated or concentrated live washed bacteria/bacterial supernatants) and the cloned, expressed, and purified recombinant protein. Both the reference virulent ST1 strain, as well as the highly virulent ST7 strain SC84 (which corresponds to the strain used in the original report by Zhang et al. [8]) presented identical results. These results also confirm the larger controversy regarding critical *S.* *suis* virulence factors [7, 43].

The study of Bek-Thomsen et al. also suggested that the *S.* *suis* Zmp homologue belongs to the ZmpC subgroup [10], and comparison of the *S.* *suis* serotype 2 Zmp sequence with that of *S.* *pneumoniae* showed 42% of homology between both proteins (Additional file 3). Moreover, the *S.* *suis* Zmp was also previously described to possess a HEMVH motif in its active site, which corresponds to that of ZmpC [8, 10]. Regardless of these genetic similarities, the specific properties and functions of the *S.* *suis* Zmp protein remained unknown. *S.* *pneumoniae* ZmpC activates the pro-form of MMP-9 and cleaves PSGL-1, MUC16, and SDC-1 ectodomains [12, 14, 15, 27].

Firstly, the involvement of the *S.* *suis* Zmp in activation of MMP-9 by pro-form cleavage was evaluated [14]. Of the different MMPs described, MMP-9 has been largely associated with the disruption of both the blood–brain barrier and the blood-cerebrospinal fluid barrier [16]. Given that one of the most important pathologies caused by *S.* *suis* in diseased pigs is meningitis, MMP-9 could play an important role in the infection [1]. Previous studies have demonstrated that *S.* *suis* serotype 2 induces production of MMP-9 from macrophages, and it was suggested that this production might help the pathogen to invade the CNS [44, 45]. Results from this study show that, under the conditions tested, and differently from *S. pneumoniae*, recombinant Zmp does not activate the pro-enzyme form of MMP-9. Moreover, we did not identify this activity in *S.* *suis* serotype 2. Thus, *S.* *suis* appears to behave similarly to *Streptococcus sanguinis* and *Streptococcus oralis*, which are also unable to activate MMP-9, despite possessing a *zmpC* gene encoding putative metalloproteases with an HEMTH motif in their active site [10]. We speculate that previously described MMP-9 activation in macrophages during interactions with *S.* *suis* may instead be due to internal control mechanisms of the host, such as the secretion of plasmin and elastase by host cells [46, 47].

PSGL-1 is an important molecule expressed at the surface of certain immune cell types and, during stress conditions, it binds the P-selectin at the surface of endothelial cells, which will subsequently allow the diapedesis of cells from the blood vessels to the site of infection [48]. Numerous pathogens, including *S. pneumoniae*, have thus developed strategies to outmaneuver the immune system, including cleavage of PSGL-1 [15, 48]. Results obtained in this study showed that, unlike that of *S.* *pneumoniae*, *S.* *suis* serotype 2 and its Zmp protein are unable to cleave PSGL-1 ectodomains.

Next, the ability of *S.* *suis* and its Zmp to cleave MUC16 was evaluated. MUC16 is a membrane-associated mucin that is part of the epithelial barrier, and it is found, among other mucosal surfaces, at the respiratory tract levels of mammals [49, 50]. *S. pneumoniae,* via its ZmpC protein, could disrupt the epithelial barrier by cleavage of MUC16 ectodomains, allowing the pathogen to more easily colonize host epithelial cells and underlying basement membranes [13, 27]. In this study, results showed that *S.* *suis* serotype 2 liberates the MUC16 ectodomains and that this function is Zmp-dependent. This could play a role in *S.* *suis* adhesion to and invasion of epithelial cells, as was shown for *Staphylococcus aureus* [51]. However, the observed activity seems to be of lower intensity that that of *S.* *pneumoniae*, as the *S.* *suis* supernatant induced this effect only after being concentrated. In addition, and differently from what was reported for *S.* *pneumoniae* [27], recombinant *S.* *suis* Zmp alone was not able to induce the cleavage of MUC16 ectodomains. Indeed, recombinant *S.* *suis* Zmp protein seems to require a co-factor found in the bacterial supernatant in order to be active. This co-factor could be a potential cation or protease: further studies are needed to examine these hypotheses in detail. In addition, *S.* *suis* has numerous virulence factors described with redundant functions that could compensate for the loss of another factor [6].

Cleavage of SDC-1 ectodomains was then investigated [12]. SDC-1 is a type 1 transmembrane heparan sulfate proteoglycan mainly expressed by plasma cells and epithelial cells and, to a lesser extent, endothelial cells, macrophages, and fibroblasts [37, 52]. SDC1 (CD138) is used as a marker for plasma cells, and myeloma cells and several carcinomas, such as breast cancer cells, express high levels of SDC1. Interactions of bacterial pathogens with heparan sulfate proteoglycans such as SDC-1 have been described as important steps in the pathogenesis of different infections [36, 53]. In accordance, the liberation of SDC-1 ectodomains by α- and β-toxins, allows *S. aureus* to avoid bacterial killing by neutrophils. This could help pathogens to persist in the circulation more easily [54]. Results obtained in the present study confirm that *S.* *suis* induces SDC-1 shedding, with this activity mainly being Zmp-dependant. Interestingly, a synergic activity was shown when Zmp protein was incubated with *S.* *suis* Δ*zmp* supernatant, supporting the hypothesis that the activity of Zmp requires a co-factor present in the *S.* *suis* supernatant. Moreover, as was observed with MUC16, a residual SDC-1 ectodomain shedding activity remains in *S.* *suis* Δ*zmp* strain, suggesting that other factor(s) could also be implicated in this function. Taken together, these functional assay results demonstrate that the *S.* *suis* serotype 2 Zmp protein does not possess all of the functions of the *S.* *pneumoniae* ZmpC.

Given that *S.* *suis* cleaved MUC16 ectodomains and the fact that the Zmp protein plays an important role in this function, its role in the adhesion to differentiated primary porcine bronchial cells was investigated. ALI culture with PBEC represents an in vitro model of well-differentiated ciliated and mucus-producing respiratory epithelial cells [29]. Since MUC16 is a mucin recovered in the mucosal barrier of respiratory epithelial cells, PBEC are a good model to evaluate the role of *S.* *suis* serotype 2 Zmp. However, both *S.* *suis* wild-type and Δ*zmp* strains had a similar capacity to adhere to PBEC under ALI conditions. This lack of difference was confirmed in vivo. Regardless of its role in MUC16 ectodomain cleavage in vitro, no difference was observed between the wild-type and the *zmp*-deficient mutant strains in colonization of tonsils and trachea by *S.* *suis* after bacterial challenge via the intranasal route. The lack of a critical role in epithelial cell adhesion and colonization might be explained by the concept of bacterial redundancy, which is very common in *S.* *suis* [7]. In fact, this result is not necessarily surprising given the number of virulence factors expressed by *S.* *suis* serotype 2 that have been reported to be implicated in colonization of the upper respiratory tracts of pigs, including adhesins and toxins. Indeed, around 40 different factors have been described to be involved in *S.* *suis* colonization [6]. Animals infected with either the wild-type or the Δ*zmp* strain also presented similar clinical signs and mortality rate. This indicates that a lower liberation of SDC-1 ectodomains by the *S.* *suis* Δ*zmp* mutant did not increase bacterial killing in vivo. As mentioned above, different anti-phagocytic bacterial factors have been described as playing important roles during *S.* *suis* infection [7].

Finally, and differently from what has been observed in a previous study [38], where the *S.* *suis* IgA protease encoded by *iga* gene was characterized as a critical virulence factor using an isogenic *iga* mutant (equivalent to the *S.* *suis* Δ*zmp* strain used herein), the *S.* *suis* Δ*zmp* strain was as virulent as the wild-type strain in an pig intranasal model of infection. In the case of *S. pneumoniae,* results differed regarding the impact of ZmpC in virulence. While a mutant Δ*zmpC* seems to have a reduced virulence in an intranasal mouse model of infection [14, 53], the Δ*zmpC* mutant showed an exacerbate virulence compared to the wild-type strain in an intravenous model of infection [55]. This does not seem to be the case for *S.* *suis*, since no significant difference was observed in virulence after intraperitoneal infection of mice with the wild-type and *zmp*-deficient mutant strains.

In conclusion, absence of an IgA_1_ protease activity in *S.* *suis* serotype 2 was confirmed. Indeed, the *S.* *suis* metalloprotease hypothetically responsible for such activity would rather belong to the ZmpC family reported for *S. pneumoniae*. The *iga* terminology for the gene coding for the Zmp of *S.* *suis* should be avoided and changed by *zmpc*. Of the different functions previously described for streptococcal ZmpC, that of *S.* *suis* would be responsible for a partial ability to cleave MUC16 and SDC-1 ectodomains. As such, the present study is the first to show such capacities for *S.* *suis*. However, the presence of this protein does not appear to be critical for colonization of the porcine upper respiratory tract. In addition, and in disagreement with published results, this protein would not be a critical virulence factor. These results further emphasize, as recently suggested, the need to confirm the critical role of reported candidates in virulence by independent laboratories [7, 43].

## Additional files

**Additional file 1.** ***S.*** ***suis*** **ST7 is unable to cleave human IgA**_**1**_. Washed bacteria (bact) or bacterial supernatant (sup) from *S.* *suis* SC84 (ST7) or *S.* *pneumoniae* (SP) were incubated with human IgA_1_ for 16 h and reactions separated by SDS-PAGE. Cleaved IgA_1_ (red arrow) by washed *S.* *pneumoniae* bacteria (lane 3) and uncleaved IgA_1_ (black arrow) by media (lane 2), washed *S.* *suis* bacteria or supernatant (lanes 4 and 5) were visualized using a specific antibody against human IgA_1_. MW corresponds to the molecular weight ladder (lane 1).

**Additional file 2.** **Zmp is not implicated in the virulence of** ***S. suis*** **serotype 2 in mouse models of infection.** Survival of six-week-old CD-1 mice infected with the wild-type strain P1/7 (WT) or its Δ*zmp* mutant via (A) the intranasal or (B) the intraperitoneal route of infection. No significant differences were observed between groups.

**Additional file 3.** ***S.*** ***suis*** **Zmp and** ***S.*** ***pneumoniae*** **ZmpC amino acid sequence alignment.** Alignment was performed using T-Coffee. Conserved amino acid appear in gray and identical amino acid appear in black.

## Abbreviations

ALI: air–liquid-interface
BCA: bicinchoninic acid
BEBM: bronchial epithelial cell basal medium
BEGM: bronchial epithelial growth medium
CPEC: circular polymerase extension cloning
DMEM: Dulbecco’s modified eagle medium
HRP: horseradish peroxidase
IgA: immunoglobulin A
IPTG: isopropyl-β-d-thiogalactopyranoside
LB: Luria-Bertani
MMP-9: matrix metalloproteinase 9
MUC16: mucin 16
PBEC: porcine bronchial epithelial cells
PBS: phosphate-buffered saline
PCR: polymerase chain reaction
pi: post-infection
PMA: phorbol-12-myristate-13-acetate
PSGL-1: P-selectin glycoprotein ligand-1
SDC-1: syndecan-1
SEM: standard error of the mean
ST: sequence type
THA: Todd-Hewitt agar
THB: Todd-Hewitt broth
WT: wild-type
Zmp: zinc metalloprotease
