# Supplementary figures and images for: Characterization of the zinc metalloprotease of Streptococcus suis serotype 2
Source: Vet Res. 2018 Oct 29;49:109. doi: 10.1186/s13567-018-0606-y (PMC6206940; doi:10.1186/s13567-018-0606-y)

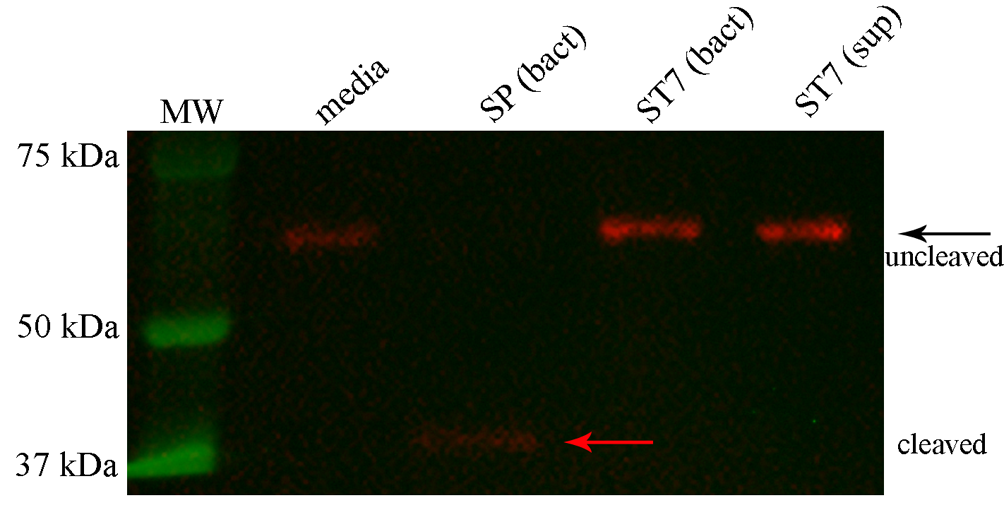

Supplement: Supplementary file 1 — Additional file 1. S. suis ST7 is unable to cleave human IgA1. Washed bacteria (bact) or bacterial supernatant (sup) from S. suis SC84 (ST7) or S. pneumoniae (SP) were incubated with human IgA1 for 16 h and reactions separated by SDS-PAGE. Cleaved IgA1 (red arrow) by washed S. pneumoniae bacteria (lane 3) and uncleaved IgA1 (black arrow) by media (lane 2), washed S. suis bacteria or supernatant (lanes 4 and 5) were visualized using a specific antibody against human IgA1. MW corresponds to the molecular weight ladder (lane 1). [file 13567_2018_606_MOESM1_ESM.tif]
