# Supplementary material for: Characterization of the zinc metalloprotease of Streptococcus suis serotype 2
Source: Vet Res. 2018 Oct 29;49:109. doi: 10.1186/s13567-018-0606-y (PMC6206940; doi:10.1186/s13567-018-0606-y)
Supplement: Supplementary file 3 — Additional file 3. S. suis Zmp and S. pneumoniae ZmpC amino acid sequence alignment. Alignment was performed using T-Coffee. Conserved amino acid appear in gray and identical amino acid appear in black. [file 13567_2018_606_MOESM3_ESM.pdf]

```
Zmp     1 MKR-SLGEKRQRFGFRKMSVGLVSAAVTSLFFVSSVATAPTAS--AQSINYSYVTEQELT
ZmpC    1 MSRKSIGEKRHSFSMRKLSVGLVSVTVSSFFLMSQGIQSVSADNMESPIHYKYMTEGKLT

Zmp    58 DGEKELIIRDLPGLAQETDVNYYLIYRPATGTTSTPSTSTSQVLPNTGSVET--ELLVAG
ZmpC   61 DEEKSLLVEALPQLAEESDDTYYLVYRS------------QQFLPNTGFNPTVGTFLFTA

Zmp   116 GVSLLLLAVRFGKKGKKELAGVILLTATGASFFGPTSSALTSQILARYNHAIEISAGQAL
ZmpC  109 GLSLLVLLVSKRENGKKRLVHFLLLTSMGVQLLPASAFGLTSQILSAYNSQLSIGVGEHL

Zmp   176 PAPAEIDGYVYVGYLKDSKAIEQTTSEEEKTAEFPASEGIRTETIVNKTEAIPFEIQTVE
ZmpC  169 PEPLKIEGYQYIGYIKTKK---QDNTELSRTVDGKYSAQRDSQPNSTKTSDVVH----SA

Zmp   236 NPQLSAGTERVVQEGQ-DGERIVTIKQVHSKGQIISEEEISSTVTKTAVPKIVEVGTKQA
ZmpC  222 DLEWNQGQGKVSLQGEASGDDGLSEKSSIAADNLSSNDSFASQVEQ---------NPDHK

Zmp   295 TDDIVTEVPDTEPSYENSTNSLTEETVTHTEVFAF-DVQEIYDVNL-AEGSREVEQKGQD
ZmpC  273 GESV---VRPTVPEQGNPVSATTVQSA-EEEVLATTNDRPEYKLPLETKGTQEPGHEGEA

Zmp   353 GVRT---IETRNYYADGVLIKSEQVSDVV-TKEPVTEVVRVGTKTTDVIGSETIVTTEEL
ZmpC  329 AVREDLPVYTKPLETKGTQGPGHEGEAAVRE-EEPAYTEPLATKGTQE------------

Zmp   409 PFETTVTETEELYVGEEKFITEGKVGSKEVTTTYQT---INGVSQPNPTVTEKVLEEPTT
ZmpC  376 ------------------PGHEGKATVREETLEYTEPVATKGTQEPEHEGEAAVEEELPA

Zmp   466 KEVLKGTKPIEGTEIETNQVEITFETEYVDDPTLLEGKTKVVTAGVNGSKTVTTTYQTIK
ZmpC  418 LEVTT--------RNRTEIQNIPYTTEEIQDPTLLKNRRKIERQGQAGTRTIQYEDYIVN

Zmp   526 GVRQENSTVTEEITKQPVKQVIARGTKVEKVPQVIITDLVENDDAKSATISYKLTDETAN
ZmpC  470 GNVVETKEVSR-TEVAPVNEVVKVGTLVKVKPTVEITNLTKVENKKSITVSYNLIDTTSA

Zmp   586 FQRAVALLYDNTGALVQEQTITDPNGQLTLENLDFYTDYTVKTKIFYTMAEQEQSSEQEA
ZmpC  529 YVSAKTQVFHG-DKLVKEVDIENPAKEQVISGLDYYTPYTVKTHLTYNLGENNEEN----

Zmp   646 ILESMRKFDLVYKKIEIKDIDAVTVYRRKNGSYIGQEFLEELPASTDELFIKVTSDRFKE
ZmpC  584 TETSTQDFQLEYKKIEIKDIDSVELYGKENDRYRRYLSLSEAPTDTAKYFVKVKSDRFKE

Zmp   706 VYLPVSSIEETTLNGKAVFKLVSSFDELVQDKDAQYVANREFYIPKM-ATDANTYTSFKA
ZmpC  644 MYLPVKSITENT---DGTYKVTVAVDQLVEEGTDGYKDDYTFTVAKSKAEQPGVYTSFKQ

Zmp   765 LLDAMKANPSGTFKLGAHLDASEVPVGDV-ASYVTN-FSGTLDGLNDGYAFSISNLKAPL
ZmpC  701 LVTAMQSNLSGVYTLASDMTADEVSLGDKQTSYLTGAFTGSLIGSDGTKSYAIYDLKKPL

Zmp   823 FFNLGG-KVQNLDIKNASLNTSSKNPLATIAINANGATITNVAVEASIKGPQNVSGLVHS
ZmpC  761 FDTLNGATVRDLDIKTVSA--DSKENVAALAKAANSANINNVAVEGKISGAKSVAGLVAS

Zmp   882 ATNTTIKGVSFKGSIEV--TGTNASLTGGILGNG--TMASVGNAKVDATITLP-GTENQV
ZmpC  819 ATNTVIENSSFTGKLIANHQDSNKNDTGGIVGNITGNSSRVNKVRVDALISTNARNNNQT

Zmp   937 AGGIVGRTMLVYDVPGSVYNSYAAGSIVTTESAAIVGGIAGANQVTGAYAPHSGNVNNVV
ZmpC  879 AGGIVGRLE----NGALISNSVATGEIRNGQGYSRVGGIVGSTW-------QNGRVNNVV
```

```
Zmp    997 SDM-TGTKSIIGQPANPTGKIKDGFTTTSDSLT--NVTVITDEEAQAKVEAMAIQATIDD
ZmpC   928 SNVDVGDGYVITGDQYAAADVKNASTSVDNRKADRFATKLSKDQIDAKVADYGITVTLDD

Zmp   1054 S-VPLNPNHYSVDYLTLDKAQADHETAYYNMEKILPFYNKELLVYYGNKIATDDKLNKVR
ZmpC   988 TGQDLKRNLREVDYTRLNKAEAERKVAYSNIEKLMPFYNKDLVVHYGNKVATTDKLYTTE

Zmp   1113 LLDVVPMKDNAVVADVYAEKANINKIMLHYADGTVDYKTVSYLEDFKNNHVVEYTISGTD
ZmpC  1048 LLDVVPMKDDEVVTDINNKKNSINKVMLHFKDNTVEYLDVTFKENFINSQVIEYNVTGKE

Zmp   1173 LIYTPESFLNDRSALVNDLVSSLSSVVLDSDAMKAVINYPTTLNADTQTGTAKDFYFGES
ZmpC  1108 YIFTPEAFVSDYTAITNNVLSDLQNVTLNSEATKKVLGAAN-------DAALDNLYLDRQ

Zmp   1233 YDQVMTNLESNVRKILV--ASLNGQGQASEDYIKEKIINNKAAFVLGLTYLNRWYDINFG
ZmpC  1161 FEEVKANIAEHLRKVLAMDKSINTTGDGVVEYVSEKIKNNKEAFMLGLTYMNRWYDINYG

Zmp   1291 EMNTKDLTIFEPDFFGNDAASALDMILAIGNGGYDVLRAHNNVTTFASIIGKQNNQTKLF
ZmpC  1221 KMNTKDLSTYKFDFNGNNETSTLDTIVALGNSGLDNLRASNTVGLYANKLASVKGEDSVF

Zmp   1351 DMLEDYRQLFLPEMTNNEWFKQTTKAYVVEGKSLIPEVAAKQETTDTYSKYNVGAYSKIV
ZmpC  1281 DFVEAYRKLFLPNKTNNEWFKENTKAYIVEMKSDIAEVREKQESPTADRKYSLGVY----

Zmp   1411 NDTVSNPTWKYNHMLLPLLTLPQENIFIITNMNTIAIGSYEHYVDDYSTV-----ENRDK
ZmpC  1337 -DRISAPSWGHKSMLLPLLTLPEESVYISSNMSTLAFGSYERYRDSVDGVILSGDALRTY

Zmp   1466 VRQMVDLAAERQRDNADFWYKILDETNRDKLFRSVLNNEGYVMYGKDGTKSYRNLT-ADV
ZmpC  1396 VRNRVDIAAKRHRDHYDIWYNLLDSASKEKLFRSVIVYDGFNVKDETGRTYWARLTDKNI

Zmp   1525 DAIQDFYGPINKWYREHPSIKTAFADGSETYYITYDMLTDYGTALYTHEMVHNQDGDIYL
ZmpC  1456 GSIKEFFGPVGKWY-EYNSSAGAYANGSLTHFVLDRLLDAYGTSVYTHEMVHNSDSAIYF

Zmp   1585 KGYGRRIGQGMEVYAQGLLQNVFNVTEMNLGFNAVYNS--DDANRVHVGDPVARFNSEAD
ZmpC  1515 EGNGRREGLGAELYALGLLQSVDSVNSHILALNTLYKAEKDDLNRLHTYNPVERFDSDEA

Zmp   1643 FNEYYHNQFDVLYLLDYLEGTNILAQSDANKKAWLRKIENYYVQNN--GVDTHAGNSARA
ZmpC  1575 LQSYMHGSYDVMYTLDAMEAKAILAQNNDVKKKWFRKIENYYVRDTRHNKDTHAGNKVRP

Zmp   1701 LTDAEVASLKSFNDLIDQSIIVQRQYVNNPTNTSKKWDRNSYVSVPMFAANFSALSNSNG
ZmpC  1635 LTDEEVANLTSLNSLIDNDIINRRSY-----DDSREYKRNGYYTISMFSPVYAALSNSKG

Zmp   1761 SPGDIMFRRMAFELIAAKGYTDGFVPYASGQLSDLAMEKGSIIYDTWNKKNTGLITDDHV
ZmpC  1690 APGDIMFRKIAYELLAEKGYHKGFLPYVSNQYGAEAFASGSKTFSSWHGRDVALVTDDLV

Zmp   1821 LEYVFQGQYTSWAEFKKAMFTERLEKAATGQLKPFTMQYELGVADSTKEVTITSFEQLQN
ZmpC  1750 FKKVFNGEYSSWADFKKAMFKQRIDKQ--DNLKPITIQYELGNPNSTKEVTITTAAQMQQ

Zmp   1881 LMKEAMEADIQAN---SLNLNNSRVHALKVKVYQALMNSTNDFRTSIFN
ZmpC  1808 LINEAAAKDITNIDRATSHTPASWVHLLKQKIYNAYLRTTDDFRNSIYK
```
